# Endotoxaemia is common in children with *Plasmodium falciparum* malaria

**DOI:** 10.1186/1471-2334-13-117

**Published:** 2013-03-05

**Authors:** Peter Olupot-Olupot, Britta C Urban, Julie Jemutai, Julius Nteziyaremye, Harry M Fanjo, Henry Karanja, Japhet Karisa, Paul Ongodia, Patrick Bwonyo, Evelyn N Gitau, Alison Talbert, Samuel Akech, Kathryn Maitland

**Affiliations:** 1 Department of Paediatrics, Mbale Regional Referral Hospital, Mbale, Uganda; 2 KEMRI-Wellcome Trust Research Programme, PO Box 230, Kilifi 80108, Kenya; 3 Liverpool School of Tropical Medicine, Molecular and Biochemical Parasitology Group, Liverpool, L3 5QA, UK; 4 Wellcome Trust Centre for Clinical Tropical Medicine, Imperial College, London, W2 1PG, UK

**Keywords:** Plasmodium falciparum malaria, African children, Severe malaria, Shock, Sepsis, Intestine, Gut-barrier dysfunction, Endotoxin, Lipopolysaccharide, Cytokines

## Abstract

**Background:**

Children presenting to hospital with recent or current *Plasmodium falciparum* malaria are at increased the risk of invasive bacterial disease, largely enteric gram-negative organisms (ENGO), which is associated with increased mortality and recurrent morbidity. Although incompletely understood, the most likely source of EGNO is the bowel. We hypothesised that as a result of impaired gut-barrier function endotoxin (lipopolysaccharide), present in the cell-wall of EGNO and in substantial quantities in the gut, is translocated into the bloodstream, and contributes to the pathophysiology of children with severe malaria.

**Methods:**

We conducted a prospective study in 257 children presenting with malaria to two hospitals in Kenya and Uganda. We analysed the clinical presentation, endotoxin and cytokine concentration.

**Results:**

Endotoxaemia (endotoxin activity ≥0.4 EAA Units) was observed in 71 (27.6%) children but its presence was independent of both disease severity and outcome. Endotoxaemia was more frequent in children with severe anaemia but not specifically associated with other complications of malaria. Endotoxaemia was associated with a depressed inflammatory and anti-inflammatory cytokine response. Plasma endotoxin levels in severe malaria negatively correlated with IL6, IL10 and TGFβ (Spearman rho: TNFα: r=−0.122, p=0.121; IL6: r=−0.330, p<0.0001; IL10: r=−0.461, p<0.0001; TGFβ: r=−0.173, p<0.027).

**Conclusions:**

Endotoxaemia is common in malaria and results in temporary immune paralysis, similar to that observed in patients with sepsis and experimentally-induced endotoxaemia. Intense sequestration of *P. falciparum*-infected erythrocytes within the endothelial bed of the gut has been observed in pathological studies and may lead to gut-barrier dysfuction. The association of endotoxaemia with the anaemia phenotype implies that it may contribute to the dyserythropoesis accompanying malaria through inflammation. Both of these factors feasibly underpin the susceptibility to EGNO co-infection. Further research is required to investigate this initial finding, with a view to future treatment trials targeting mechanism and appropriate antimicrobial treatment.

## Background

Endotoxin is a complex lipopolysaccharide (LPS) present in the cell walls of gram-negative bacteria and in substantial quantities in the bowel, and a potent trigger of innate immunity due to activation of Toll Like Receptor (TLR) signalling [1]. The effects of endotoxin in sepsis are wide ranging, including upregulation of endothelial cytoadherence receptors [2], anaemia and various metabolic disturbances such as acidosis and hypoglycaemia. Endotoxin acts as a potent stimulus for the release of pro-inflammatory cytokines such as TNFα, IL1 and IL6 that have been implicated in the pathophysiology of severe infectious diseases, including malaria. Nevertheless, establishing the relationship between endotoxaemia and infection has been frustrated by the limitations of available endotoxin assays [3]. In adults with critical illness, recent studies have shown that endotoxaemia is associated with mortality, organ failure and prolonged intensive care unit admission [4]. In children, endotoxaemia has been associated with more severe illness requiring admission to intensive care, severity or duration of illness but not mortality [5,6].

Severe forms of *Plasmodium falciparum* malaria are characterised be excessive inflammation, organ damage and metabolic dysregulation, a picture similar to that observed in patients suffering from severe bacterial sepsis [7]. Malaria infection strongly predisposes African children to invasive bacterial disease (IBD). Whilst the epidemiological data supporting this causal link are robust the mechanism for this association remains obscure. The leading causes of IBD in *P. falciparum* infection are non-typhoidal Salmonellae (NTS), *E. coli* and other enteric organisms with gram-negative organisms (EGNO) becoming increasingly more predominant across the severity spectrum: from those with evidence of recent malaria infection (slide negative, Paracheck-Pf**®** positive), intercurrent malaria and greatest in those with severe malaria and hyperparasitaemia [8-12]*.* Although the mechanisms underlying the increased susceptibility are uncertain, they may include disordered gut barrier function secondary to intense sequestration of parasitized red cells in the intestine [13-15] and/or acute severe perfusion injury secondary to severe anaemia (haemoglobin < 5 g/dl) and shock. A recent study in a rodent model of malaria showed that induction of heme oxygenase I in neutrophils during acute malarial anaemia resulted in loss of function and was associated with increased susceptibility of mice to *Salmonella typhimurium* bacteraemia [16]. These observations have now been corroborated in children with malaria showing that neutrophil dysfunction was associated with parasite biomass and markers of hemolysis [17].

We hypothesised that either translocation of endotoxin alone or translocation of gram-negative bacteria and subsequent release of endotoxin into the blood stream may play a role in pathogenesis of severe malaria and its complications such as shock and organ dysfunction. We prospectively examined endotoxin levels and its relationship to disease severity and cytokine activation in children with malaria at two hospitals in East Africa.

## Methods

### Study populations

Between August and December 2009 paediatric admissions to Kilifi District Hospital (KDH), Kenya with *P. falciparum* malaria and the acute paediatric ward at the Mbale Regional Referral Hospital (MRRH), Eastern Uganda were recruited. In Kilifi District malaria transmission is now mesoendemic due to a steady decline in transmission over the last 10 years [18]. In Mbale, malaria occurs throughout the year and is consistent with high stable transmission [19].

Three groups were considered based on the severity of clinical symptoms: First, children hospitalised with *P. falciparum* malaria with signs of severe illness but without life-threatening clinical syndromes (n=51). Second, children with severe, life-threatening malaria (n=206) included children with respiratory distress (deep breathing or indrawing), impaired consciousness (prostration or coma) or severe anaemia (haemoglobin, Hb ≤5g/dl) [20]. The severe group was further subdivided into those with (n=154) or without (n=52) shock (defined as at least one of: severe tachycardia, delayed capillary refill (>2s), temperature gradient or weak pulse) [21]. A proportion of children (23 in Kilifi, 100 in Mbale) were recruited as part of the FEAST study (Fluid Expansion as a Supportive Therapy) [21]. The third group, included 25 healthy children who returned to KDH for a follow-up appointment four weeks after a severe malaria episode (healthy control group). Clinical examination showed that these children were afebrile and free of malaria parasites by microscopy.

Malaria was diagnosed by a rapid malaria test (DiaMed OptiMAL®) and a blood film. In Mbale, parasite blood films were screened for the presence of parasites but parasite densities not reported whereas parasite densities were reported in Kilifi. Clinical history, presentation and laboratory values where recorded for all children using the FEAST Case Report Form. Blood cultures were done on all study participants in KDH but not in MRRH. At KDH, only two children were blood culture positive whereas in all other children no growth was reported. Children presenting with severe anaemia (haemoglobin ≤5g/dl) were transfused 20mls/kg whole blood over 3 h. Moderate anaemia was defined as an Hb>5 and ≤7g/dl. All children received parenteral antimalarials and antibiotics.

The study was approved by the Ugandan Ethical Review Committee (REIRC 002/2009) and the National Ethical Review Committee in Kenya (SSC nos. 1135 and 1247). Parents or guardians of all children recruited to take part in the study provided written informed consent.

### Endotoxin assay

We used an Endotoxin Activity Assay (EAA™ Spectral Diagnostics) to detect the presence of endotoxin in whole blood within 3 h of venepuncture according to manufacturers instructions. This is a novel chemiluminescent assay that relies on the detection of Lipid A component of lipopolysaccharide (LPS), which is not susceptible to microbial contamination. It measures endotoxin activity in whole blood by the priming of host neutrophil respiratory burst activity via complement opsonised LPS-IgM immune complexes and has shown good correlation with Gram-negative infection and with clinical sepsis, which was not apparent using limulus amoebocyte lysate (LAL) assay [3,4]. The cut off value for clinically relevant endotoxaemia, evaluated in the MEDIC trial was 0.4 EAA Units (equivalent to an endotoxin concentration of 25–50 pg/ml of *Escherichia coli* 055:B5 LPS) with high endotoxin levels (at or above 0.6 EAA Units, equivalent to 200 pg/ml of *Escherichia coli* 055:B5 LPS) related to prognosis [4].

### ELISA

Plasma was separated and stored in aliquots at -80C. The cytokine concentration of TNFα, IL6, IL10, and TGFβ was determined using Quantikine ELISA Kits (R&D) according to the manufacturer’s instructions. The plasma concentration of the intestinal fatty acid binding protein (I-FABP) [22] was determined using an ELISA kit from HyCult. The detection limits for all cytokines were at or below 20 pg/ml.

### Statistical Analysis

The presence or absence of clinical parameters in the different study groups were determined using Pearson χ^2^. Continuous values such as age and cytokine concentrations were compared between groups using the Mann Whitney *U* test or Kruskal Wallis H test. Spearman’s rho correlation coefficient was used to determine a relationship between continuous variables. Statistical analyses were performed using SPSS 18 (IBM SPSS Statistics) and Stata version 11 (StataCorp LP).

## Results

### Clinical presentation of children with malaria

We recruited 257 children, 205 at MRRH and 52 at KDH, the basic clinical presentation of all children is described in Table 1. In the group with severe malaria, Kenyan children were older, had a higher prevalence of impaired consciousness and a lower prevalence of severe anaemia or respiratory distress compared to children recruited from MRRH, as expected in areas of lower transmission intensity (Table 2). Blood culture data were available for children admitted to KDH but only 2 cases were blood culture positive. The only fatalities occurred in the severe malaria cases with shock.

**Table 1:** Baseline clinical features of children with malaria

	**Non-severe malaria**	**Severe malaria**	**Severe malaria & shock**	**P**
**Age (months)**^**a**^	30.2 (15.5-52.8)	32.5 (14.8-57.6)	31.9 (14.8-48.2)	0.726
**Impaired perfusion**^**b**^	31.4% (16/51)	-	100% (154/154)	<0.0001
**Respiratory distress**^**b**^	-	71.2% (37/52)	83.1% (128/154)	0.049
**Impaired consciousness**^**b**^	-	36.5% (19/52)	71.4% (110/154)	<0.0001
**Coma**^**b**^	-	9.6% (5/52)	20.8% (32/154)	0.067
**Severe anaemia**^**b**^	-	11.8% (6/51)	34.7% (52/150)	0.002
**Mortality (48 h) **^**b**^	-	0% (0/52)	6.5% (10/154)	-
**Endotoxaemia (EAA**≥**0.4) **^**b**^	31.4% (16/51)	28.3% (15/52)	26.0% (40/154)	0.738
**High endotoxaemia (EAA**≥**0.6) **^**b**^	9.8% (5/51)	5.8% (3/52)	10.4% (16/154)	0.608

^a^ Shown are median and in parenthesis 25th and 75th percentile. For comparison of difference in age between groups, Kruskal-Wallis H test was used.

^b^ Percentage of children presenting with syndrome and in parenthesis the number of children presenting with a syndrome over the total number of children in that group. For comparison of the proportion of children presenting with a given syndrome, Pearson χ^2^ was used.

**Table 2:** Spectrum of clinical presentations of severe malaria in children in Kilifi, Kenya and Mbale, Uganda

	**KDH**	**MRRH**	**P**
**Age (months)**^**a**^	48.5 (37.6-70.7)	24 (13.1-42.3)	<0.0001
**Impaired perfusion**^**b**^	79.2% (38/48)	73.2% (115/157)	0.410
**Respiratory distress**^**b**^	45.8% (22/48)	91.1% (143/157)	<0.0001
**Impaired consciousness**^**b**^	91.7% (44/48)	54.1% (85/157)	<0.0001
**Coma**^**b**^	31.3% (15/48)	14% (22/157)	0.007
**Severe anaemia**^**b**^	14.6% (7/48)	32.9% (50/152)	0.014
**Mortality (48 h)**^**b**^	4.2% (2/48)	5.1% (8/157)	0.794
**Endotoxaemia**^**b**^	12.5% (6/48)	31.2% (49/157)	0.01
**High endotoxaemia**^**b**^	2.1% (1/48)	11.5% (18/157)	0.05

^a^ Shown are median and in parenthesis 25th and 75th percentile. For comparison of difference in age between groups, Kruskal-Wallis H test was used.

^b^ Percentage of children presenting with syndrome and in parenthesis the number of children presenting with a syndrome over the total number of children in that group. For comparison of the proportion of children presenting with a given syndrome, Pearson χ^2^ was used. Differences in mortality were not calculated because all cases occurred in the group of children with severe malaria and shock.

### Children with malaria have elevated concentration of endotoxin in their blood

Endotoxaemia (EAA≥0.4) was detected in 16 (31.4%) children with non-severe malaria, 15 (28.3%) children with severe malaria but not detected in any of the 25 healthy children recruited as a control group. The prevalence of endotoxaemia, in the subgroup of children with severe malaria and shock was 40/154 (26%), with 3/10 (30%) in fatal cases and 37/144 (25.6%) in non-fatal cases suggesting that the presence of endotoxaemia was neither associated with disease severity nor outcome.

The proportion of children with severe malaria complicated by endotoxemia was lower in the cohort recruited at KDH than in the cohort recruited at MRRH, as was the number with high endotoxin levels (Table 2). This could be due to either difference in age or transmission intensity in these cohorts. We were unable to show any relationship of age and endotoxin level within each cohort (KDH: Spearman rho=−0.225, p=0.110; MRRH: Spearman rho=−0.082, p=0.242). Nor did we find any correlation between endotoxin levels and parasite density in the 52 children with independently verified malaria parasite quantification (Spearman rho=−0.018; parasite density median (25th and 75th percentile): 183,280/ml (4,418-428,840/ml)).

### Endotoxaemia and clinical presentation

Endotoxaemia (EAA≥0.4) was more likely to be present in children with moderate anaemia (Hb>5 and ≤7g/dl) or severe anaemia (Hb≤5g/dl) compared to children without anaemia (Hb>7g/dl) (Table 3). By contrast, endotoxaemia was less common in children with impaired consciousness than in those without altered conscious level. However, this observation maybe confounded by the higher number of children presenting with impaired consciousness at KDH who overall had a lower prevalence of endotoxaemia. In order to exclude this possibility we conducted a separate analysis of children admitted to MRRH (n=205), which showed that there was a similar prevalence of endotoxaemia in children with impaired consciousness (27.1% (23/85)) compared to those without impaired consciousness (34.2% (41/120); Pearson χ^2^ = 1.17, p=0.299). We found no association between endotoxin with other clinical subgroups or with outcome.

**Table 3:** Association of severe malaria clinical subgroups and endotoxaemia

		**n with endotoxin **≥ **0.4/N with clinical feature (%)**	**χ**^**2**^	**P**^**a**^
**Moderate anaemia**	**Hb>7 g/dl**	30/151 (19.9%)		
	**Hb>5 and ≤7 g/dl**	14/40 (34.4%)	4.084	0.043
**Severe anaemia**	**Hb>7 g/dl**	30/151 (19.9%)		
	**Hb≤5 g/dl**	24/58 (41.4%)	10.12	0.001
**Any anaemia**	**Hb>7 g/dl**	30/151 (19.9%)		
	**Hb≤7 g/dl**	38/98 (38.8%)	10.7	0.001
**Impaired consciousness**	**None**	43/128 (33.6%)		
	**Prostration or Coma**	28/129 (21.7%)	4.5	0.033
**Respiratory distress**	**None**	27/92 (29.3%)		
	**Present**^**b**^	44/165 (26.7%)	0.21	0.645
**Shock**	**None**	32/106 (30.2%)		
	**Shock**	39/151 (25.8%)	0.592	0.442
**Mortality**	**Non-fatal**	68/247 (27.5%)		
	**Fatal**	3/10 (30%)	0.03	0.864

^a^ Pearson χ^2^ with 1° of freedom and corresponding P value.

^b^Deep breathing or Indrawing.

### Association of endotoxaemia and cytokine level in children with severe malaria

In critically ill patients, endotoxin acts as a potent stimulus for the release of pro-inflammatory cytokines followed by a phase of immune paralysis characterised by control of inflammation. We speculated that children with malaria and endotoxaemia could show either elevated or depressed levels of pro-inflammatory cytokines. Plasma concentrations of TNFα, IL6, IL10 and TGFβ are presented in Table 4 by subgroup and outcome. Plasma concentration of TNFα, IL6 and IL10 but not TGF β increased with increasing disease severity (Table 4). Whilst cytokine profiles were similar amongst children in different subgroups of severe malaria, IL6, was higher in the subgroup with shock (Mann Whitney *U* test, p<0.016). Fatal cases had significantly higher plasma concentrations of IL6 than those who survived (Mann Whitney *U* test p=0.022).

**Table 4:** Plasma cytokine concentrations in children with different disease severity

	**Non-severe malaria**	**Severe malaria**	**Severe malaria & shock**	**Fatal cases**	**P**^**a**^
**N**	44	30	126	10	
**TNFα (pg/ml)**^**b**^	0 (0–19.3)	0 (0–59.3)	28.8 (0–70)	0 (0–115)	0.014
**IL6 (pg/ml)**^**b**^	0 (0–20.8)	0 (0–82)	52.5 (0–219)	524 (30–1252)	<0.0001
**IL10 (pg/ml)**^**b**^	105 (0–373)	211 (59–744)	471 (135–1159)	492 (62–1924)	0.002
**TGFβ (pg/ml)**^**b**^	467 (83–1371)	453 (0–938)	510 (0–1101)	793 (0–1037)	0.499
**I-FABP (pg/ml)**^**b,c**^	0 (0–205)	0 (0–429)	0 (0–310)	218 (0–1138)	0.217

^a^Kruskal Wallis H test.

^b^Shown are median and 25^th^ and 75^th^ percentile.

^c^The plasma concentration of I-FABP was measured in 43 children with non-severe malaria, 30 children with severe malaria, 116 children with severe malarial shock and 9 fatal cases.

**Figure 1:**
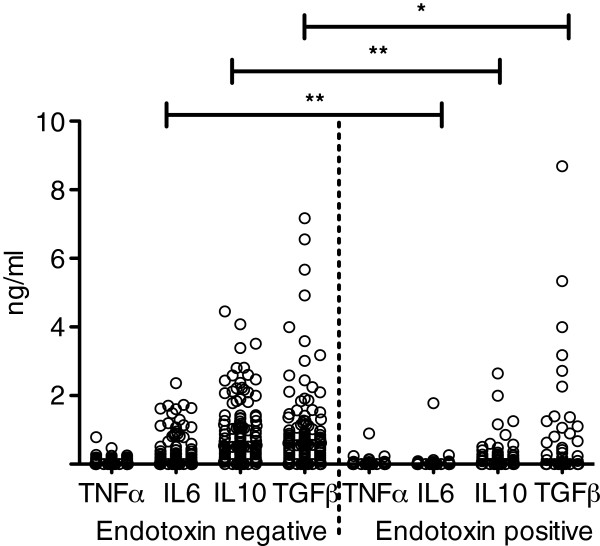
**Scatterplot of plasma cytokine concentrations in all children suffering from severe malaria with or without endotoxaemia (EAA >0.4 units).** Differences in the plasma cytokine concentration in children with or without endotoxaemia were determined using Mann Whitney U tests and significant differences are indicated with p<0.0001 for IL6 and IL10 (**) and p<0.05 for TGFβ (*). Horizontal bars represent the median.

Of note, in all subgroups of children with severe malaria plasma concentrations of IL6, IL10 and TGFβ were reduced in the presence of endotoxaemia compared to those without endotoxaemia (Figure 1). Plasma endotoxin levels in severe malaria negatively correlated with IL6, IL10 and TGFβ (Spearman rho: TNFα: r= −0.122, p=0.121; IL6: r=−0.330, p<0.0001; IL10: r=−0.461, p<0.0001; TGFβ: r=−0.173, p<0.027). Interestingly, the plasma concentration of TNFα was not modified by endotoxaemia (median TNFα (25^th^ and 75^th^ percentile) with endotoxaemia: 0 pg/ml (0–54.3) without endotoxaemia: 26.7 pg/ml (0–73.9) Mann Whitney *U* test, p=0.2).

### Intestinal fatty acid binding protein is increased in children with malaria

**Figure 2:**
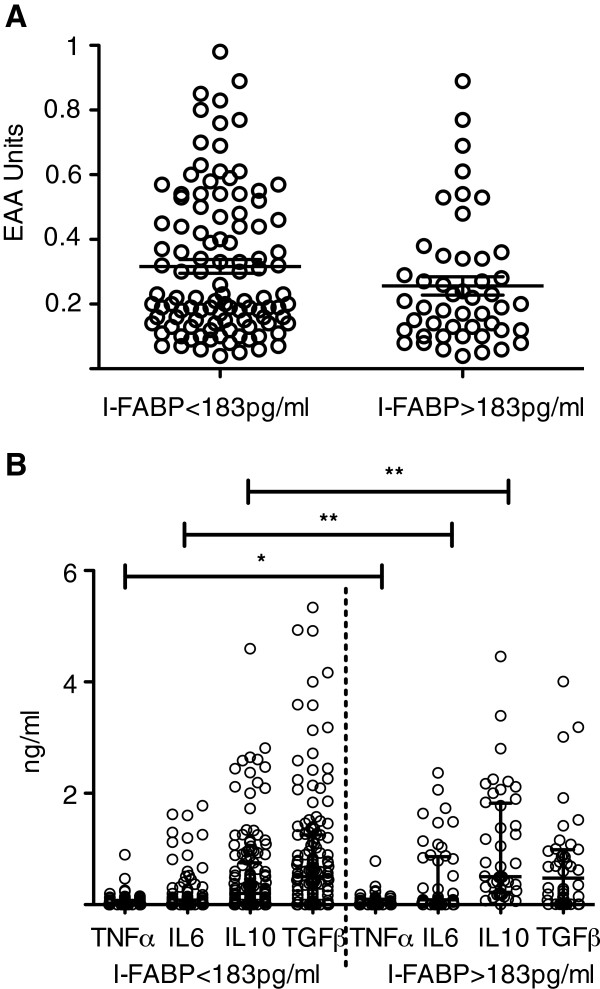
**Endotoxin levels and cytokine concentrations in children with increased plasma -concentration of I-FABP.** Shown are scatter plots of endotoxin EAA Unit (**A**) and plasma cytokine concentration (**B**) in children with malaria and plasma I-FABP concentration above or below 183 pg/ml. Differences in the plasma cytokine concentration in children with or without increased plasma I-FABP were determined using Mann Whitney U tests and significant differences are indicated with p<0.001 for IL6 and IL10 (**) and p<0.05 for TGFβ (*). Horizontal bars represent the median.

We measured the plasma concentration of the intestinal fatty acid binding protein (I-FABP), a marker for acute enterocyte death and putative intestinal permeability, in 198 samples (Table 4). Neither the proportion of children with detectable levels of I-FABP nor its median plasma concentration differed between children with or without endotoxaemia (Figure 2A). However, in children with an I-FABP plasma concentration above 183 pg/ml (Figure 2B) – a level that is currently indicated as being associated with acute intestinal injury [23] - plasma cytokine concentration of TNFα, IL6 and IL10 were significantly higher in these children compared to those without or lower concentrations of I-FABP.

## Discussion

To our knowledge this is the first study to document the prevalence of endotoxaemia in children suffering from malaria. Approximately 30% of children with malaria had detectable levels of endotoxin (LPS) in their blood. Surprisingly, and contrary to findings in other critical illnesses but not all paediatric studies [6], we found no evidence that endotoxaemia or high levels of endotoxaemia (EAA> 0.6) were associated with the degree of disease severity or led to a worse outcome. These findings suggests that endotoxaemia may be important in the transition of an uncomplicated episode of malaria in children living in endemic regions to requiring hospitalisation but does not distinguish those at greatest risk of life-threatening complications or organ dysfunction, as in adults with severe sepsis [4].

In our study, the most notable clinical parameter associated with endotoxaemia was anaemia. The origins of malaria anaemia are multiple. Next to acute vascular haemolysis of infected erythrocytes during acute malaria, both acute and chronic infection cause inflammation resulting in dyserythropoiesis due to changes in iron storage metabolism and cytokine-mediated suppression of the generation of progenitor cells [24-26]. It seems unlikely that impaired oxygen delivery to the bowel as a result of anaemia, directly causes endotoxaemia but rather that inflammation is independently associated with anaemia and increased gut permeability. Additional evidence comes from studies in patients suffering from sepsis and of endotoxaemia induced in human volunteers, which suggested that inflammation resulted in enterocyte death, increased gut permeability or both [27,28]. I-FABP is particularly highly expressed in cells present on the tips of the villi, the initial site of ischaemia reperfusion injury, indicating that circulating I-FABP is a useful plasma marker for identifying bowel injury secondary to splanchnic hypoperfusion [22]. This association has been demonstrated in both murine and human studies of ischaemia reperfusion injury [23,29]. We therefore tested the plasma concentration of I-FABP in children with and without endotoxaemia. In our study, I-FABP was detected in a similar proportion of patients with or without endotoxaemia, contrary to the findings in other studies [5]. However, we may have missed a direct association between endotoxaemia and I-FABP because the half-life of I-FABP is estimated to be 11 min [30] and plasma I-FABP concentrations reflect immediate enterocyte damage rather than recent cell damage. By contrast, pathological concentrations of I-FABP were associated with increased plasma concentrations of TNFα, IL6 and IL10 suggesting that at least in a proportion of children, enterocytes and thus the gut barrier function are damaged due to inflammation.

We hypothesised that the most likely source of endotoxaemia is the gastrointestinal tract. Gastro-intestinal symptoms are common in malaria yet there has been little recent interest in gut barrier dysfunction despite evidence provided over 2–3 decades ago demonstrating histopathological changes and impaired absorption [13,14,31,32]. Histopathological studies have demonstrated intense sequestration of *P. falciparum*-infected erythrocytes within the endothelial bed of the gut, particularly at the tip of intestinal villi [13,14] and the presence of small bowel intussusceptions in children with severe malaria [15] has been reported. The central pathological processes of falciparum malaria include increased rigidity (or non-deformability) of non-parasitized red cells [33] and intense sequestration of late stage infected erythrocytes in venules and capillaries- both of which compromise microcirculatory flow to vital organs and alter endothelial cell function [34]. Sequestration may favour direct translocation of gram-negative bacteria or, through induction of inflammation, lead to injury and an impaired gut barrier function with the transfer of endotoxin and/or pathogenic bacteria into the blood stream.

Sequestration is mediated by parasite molecules expressed on the surface of infected erythrocytes that mediate their adhesion to receptor expressed on endothelial cells [35]. Interestingly, infected erythrocytes from children suffering from severe anaemia usually show reduced adhesion to these receptors [36,37] which may increase the release of inflammatory cytokines in these patients [38] providing another link between inflammation and anaemia. In addition, during acute blood stage malaria TLR agonists such as parasite DNA and GPI are released [39,40] and may increase responsiveness of leukocytes to secondary challenge with TLR ligands, at least in uncomplicated malaria [41,42]. Both factors independently increase inflammation and may lead to inflammation-induced anaemia and loss of gut barrier function resulting in the translocation of endotoxin and/or bacteria into the blood stream.

Together these data suggest that parasite-mediated sequestration and inflammation contribute to both anaemia and changes in gut permeability. However, particularly TLR-mediated induction of inflammation leads to altered responsiveness of leukocytes to additional signals such as endotoxin-tolerance. In our study, endotoxaemia was associated with decreased plasma concentrations of TNFα, IL6 and IL10 compared to those without endotoxaemia [43]. In sepsis, it has been shown that patients may enter a phase of immune-paralysis signified by control of inflammation, alternate signalling pathways in leukocytes and enhanced apoptosis of immune effector cells [44]. Although the control of inflammatory responses reduces organ damage and early mortality, it comes at the expense of increased vulnerability to infection due to either activation of latent viruses or secondary infection with pathogens and increased risk of mortality [44-46]. Thus, endotoxin-mediated depression of inflammatory immune responses may contribute to decreased ability of malaria patients to control secondary bacterial infection.

There is strong evidence that recent or current infection with malaria increases the risk of systemic bacterial infection with high associated mortality rates in several sub-Saharan countries [8,9,11,47,48]. Of note, a study in Tanzanian children with recent or current malaria demonstrated that one of the key bedside predictors of IBD (largely NTS and other Gram-negative organisms) was severe anaemia [12]. Recent studies suggested that that the association between NTS and anaemia may be due to changes in neutrophil function as a result of changes in iron storage metabolism and subsequently inefficient control of pathogenic bacteria once they have entered the blood stream [17].

Our study is limited by the absence of blood culture data and parasite density data, which were available only for a very small number of children. All but one child with blood culture data were negative for gram-negative organisms suggesting that endotoxaemia resulted from endotoxin translocation due to gut barrier dysfunction. Despite the small number of children with blood culture data, this appears plausible since the prevalence of endotoxin observed in this study was much higher than previous reports of bacterial co-infection, which are in the order of 5-12% [8-12]; but this is confounded by the low sensitivity of blood culture and preadmission antibiotic use. Furthermore, we measured endotoxaemia and plasma cytokines only at admission of children with acute disease and therefore can only speculate on the link between endotoxaemia, inflammation and subsequent immune-paralysis. Repeat sampling from the early stages of infection through to acute illness and resolution of malaria would be required to elucidate the complex interactions of inflammatory pathways and their regulation.

## Conclusions

We suggest the following hypothesis: A proportion of children suffering from malaria experience loss of the barrier function of the gut due to parasite sequestration (and reduced microcirculatory flow), inflammation or both. This could be augmented by other pathophysiological processes, specific to falciparum malaria including severe anaemia and those leading to microvascular obstruction. In some children, loss of gut barrier function might be sufficient to allow translocation of endotoxin and translocation of gram-negative bacteria with subsequent release of endotoxin into the bloodstream. Endotoxin and other TLR–ligands expressed by gram-negative bacteria could further increase malaria-induced inflammation followed by regulatory immune responses that result in temporary immune paralysis as has been described for sepsis. Then children, having entered a phase of immune paralysis, might not be able to mount an adequate immune response controlling bacteraemia. This hypothesis is testable through investigation of gut-barrier function in children suffering from malaria, characterisation of the cytoadhesion profile of clinical parasite isolates from children with malaria and endotoxaemia and by analysing differences in the innate and adaptive immune response pathways in children with severe malaria in the presence or absence of endotoxaemia.

## Abbreviations

EGNO: Enteric gram negative organisms; NTS: Non-typhoidal Salmonellae; I-FABP: Intestinal fatty acid binding protein; KDH: Kilifi district hospital; MRRH: Mbale regional referral hospital.

## Competing interests

None of the authors report a conflict of interests.

## Authors’ contributions

Design of the study: POO, KM, BCU. Patient recruitment: JK, JN, PO, SA, AT. Laboratory analysis: HMF, HK, EG, PB. Statistical analysis: JJ, BCU. Manuscript preparation: POO, KM, BCU. All authors read and approved the final manuscript.

## Pre-publication history

The pre-publication history for this paper can be accessed here:

http://www.biomedcentral.com/1471-2334/13/117/prepub
